# Accurate and Efficient Structure Elucidation from Routine One-Dimensional NMR Spectra Using Multitask Machine Learning

**DOI:** 10.1021/acscentsci.4c01132

**Published:** 2024-11-13

**Authors:** Frank Hu, Michael S. Chen, Grant M. Rotskoff, Matthew W. Kanan, Thomas E. Markland

**Affiliations:** † Department of Chemistry, Stanford University, Stanford, California 94305, United States; ‡ Simons Center for Computational Physical Chemistry, Department of Chemistry, New York University, New York, New York 10003, United States

## Abstract

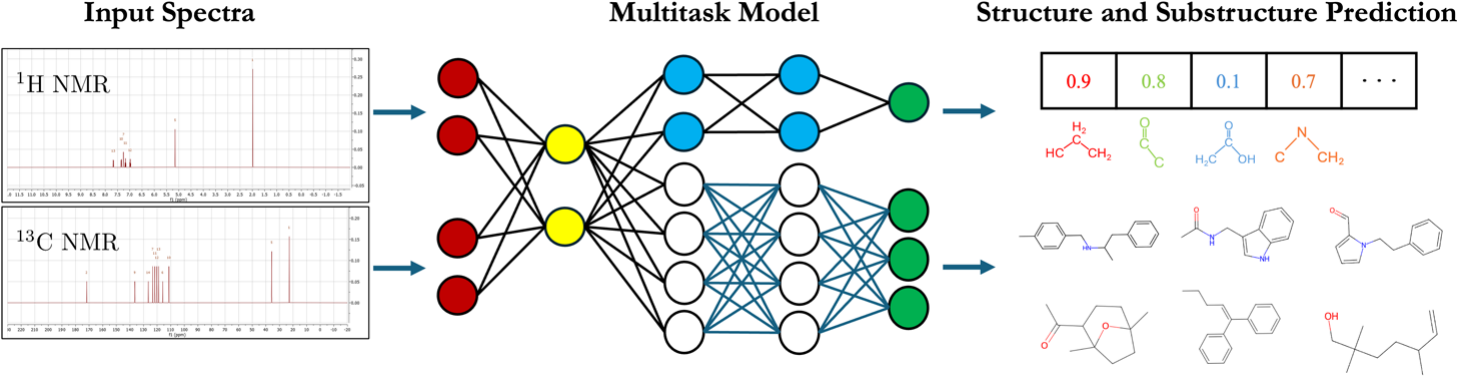

Rapid determination of molecular structures can greatly accelerate workflows across many chemical disciplines. However, elucidating structure using only one-dimensional (1D) NMR spectra, the most readily accessible data, remains an extremely challenging problem because of the combinatorial explosion of the number of possible molecules as the number of constituent atoms is increased. Here, we introduce a multitask machine learning framework that predicts the molecular structure (formula and connectivity) of an unknown compound solely based on its 1D ^1^H and/or ^13^C NMR spectra. First, we show how a transformer architecture can be constructed to efficiently solve the task, traditionally performed by chemists, of assembling large numbers of molecular fragments into molecular structures. Integrating this capability with a convolutional neural network, we build an end-to-end model for predicting structure from spectra that is fast and accurate. We demonstrate the effectiveness of this framework on molecules with up to 19 heavy (non-hydrogen) atoms, a size for which there are trillions of possible structures. Without relying on any prior chemical knowledge such as the molecular formula, we show that our approach predicts the exact molecule 69.6% of the time within the first 15 predictions, reducing the search space by up to 11 orders of magnitude.

## Abstract 2

We introduce a multitask machine learning framework that rapidly predicts both the molecular structure and molecular fragments of an unknown compound using only one-dimensional ^1^H and ^13^C NMR spectra.

## Introduction

1D ^1^H and ^13^C NMR spectroscopy are workhorse methods in structure elucidation, since they are both easy to perform and provide a wealth of information about molecular composition, connectivity, and stereochemistry. However, interpretation of NMR spectra remains time-intensive and error-prone, often requiring high levels of chemical expertise and prior knowledge that constrains the space of possible structures. For small molecules or structures that are composed of a known subset of chemical building blocks, the measured spectra can be compared with databases of spectra, obtained either from previous experiments or forward prediction methods,^1−4^ with the hope of obtaining a match.^5−13^ However, the number of possible molecules suffers from a combinatorial explosion as the number of constituent atoms increases. For example, for organic compounds made up of up to 11 heavy (non-hydrogen) atoms (C, N, O, S, and/or halogen), there are ∼26 million molecules consistent with the basic bonding rules of chemistry (not including stereoisomers), but by 17 heavy atoms, this number grows to ∼166 billion possible structures,^14^ and by 21 heavy atoms, exceeds 20 trillion. Addressing this formidable challenge to unlock unsupervised structure elucidation of organic molecules would remove a key bottleneck in chemical research and could be paired with automated synthetic workflows^15−17^ to create closed-loop discovery platforms.

We and others have recently reported machine learning (ML) approaches to structure elucidation based on identifying molecular substructures (functional groups, small fragments) from NMR spectral data and using this information to assemble a molecular structure^18−20^ or based on the task of selecting which of a set of provided candidate molecules corresponds to a given NMR spectrum and assigning the peaks in that provided spectrum to specific atoms.^21^ While ML models have proven to be remarkably effective in identifying substructures, converting substructure information to a molecular structure is much more challenging. Beam searching over possible structures^20^ or building the structure in an atom-by-atom manner^22,23^ are viable for small systems but quickly lose effectiveness as the number of atoms increases because of the combinatorial scaling of the problem size.

Rather than predicting fragments as an intermediate step, recent work has sought to directly predict molecular structure from spectra in an end-to-end fashion, using deep learning architectures such as graph convolutional neural networks^24^ and transformers.^25−27^ For molecules with up to 13 heavy atoms a top-10 accuracy of 78.5% was achieved with a transformer model using as inputs the molecular formula and infrared (IR) spectra that were preprocessed into a sequence of integers representing the intensity.^27^ On systems of up to 35 heavy atoms, a similar approach with transformers yielded a top-10 accuracy of 86.6% using the molecular formula and ^1^H NMR and ^13^C NMR spectra preprocessed into strings containing information about peak shifts, splittings, and multiplicities.^26^ For larger organic systems ranging from a few dozen to over a hundred heavy atoms, it was shown that one can obtain a top-10 accuracy of 94.2% when feeding the molecular formula, ^13^C NMR shifts, and a SMILES^28^ string representation of a large fragment of the target molecule into a transformer pretrained on 360 million molecules.^25^ These studies suggest that end-to-end frameworks are a promising approach to address the combinatorial problem when extensive preprocessing is applied to the spectra and sufficient chemical information is provided (e.g., the molecular formula). However, in many cases, information such as the molecular formula is not readily available, and preprocessing spectra is burdensome and introduces biases. It is, therefore, critical to develop a framework that can accurately and efficiently predict the molecular structure of an unknown compound using raw spectral data alone.

Here, we present a transformer-based ML framework for solving the most challenging version of the structure elucidation problem, applying minimal preprocessing to the input ^1^H and ^13^C NMR spectra and using no other prior information such as molecular formula or molecular fragments. First, we train a transformer model^29^ to solve the problem of constructing the molecular structure (formula and connectivity) when given only information about the presence or absence of a set of 957 very simple substructures (≤7 atoms). We show that a transformer architecture recovers the exact molecular structure with high accuracy, succeeding 93.2% of the time within the first 15 predictions when tested on molecules with up to 19 heavy atoms. Next, we integrate this pretrained transformer into a multitask model that predicts both substructure and molecular structure. This end-to-end model inputs only 1D ^1^H NMR and ^13^C NMR spectra and yields the correct molecular structure 69.6% of the time within the first 15 predictions when tested on simulated spectra for molecules with up to 19 heavy atoms. This model is thus capable of rapidly constraining the massive chemical search space (>2 trillion possibilities) using just routinely available NMR spectra, providing a complementary tool to other existing structure elucidation, reaction prediction, and retrosynthesis frameworks.

## Results and Discussion

**Figure 1:**
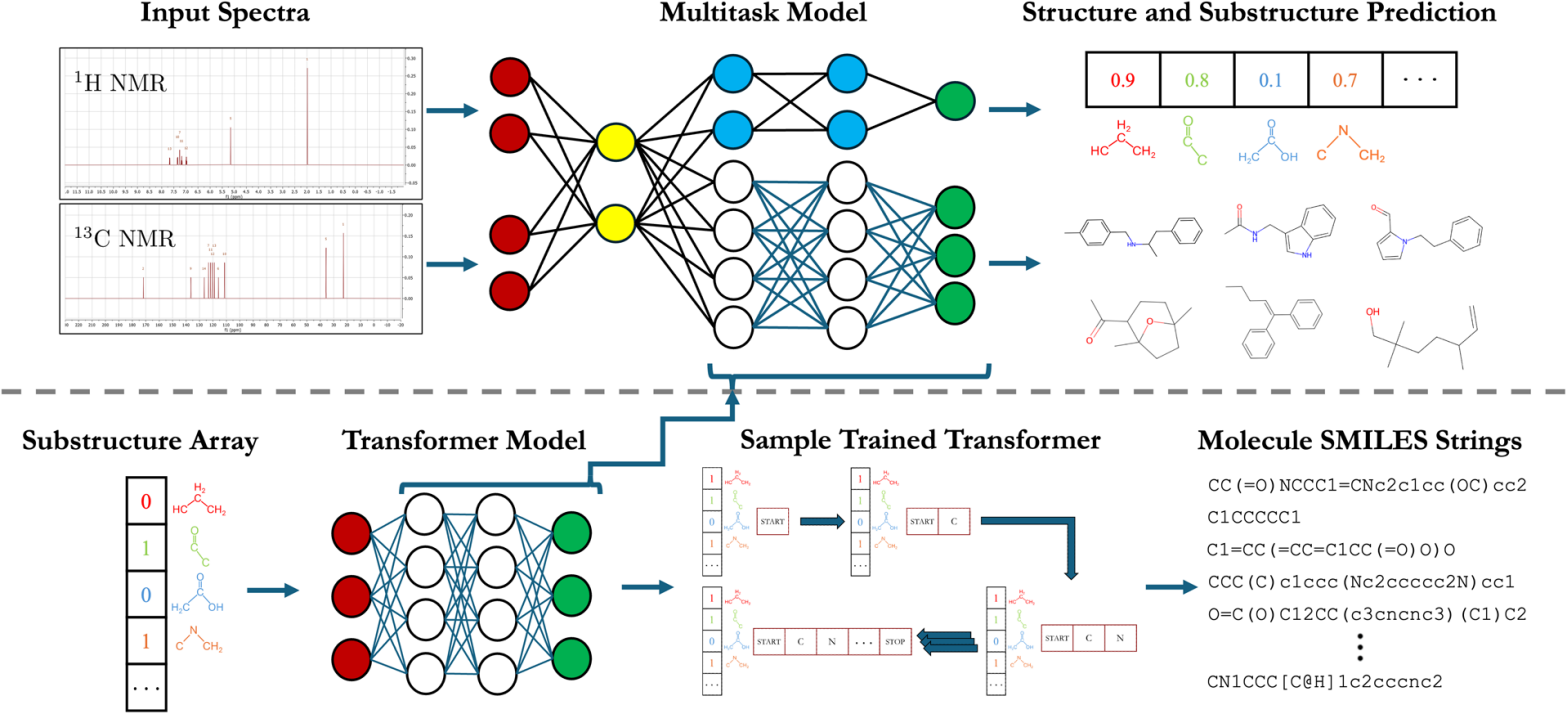
Overview of the full multitask structure elucidation workflow (top) and the substructure-to-structure workflow (bottom). Weights from a transformer pretrained on the substructure-to-structure task are used to initialize the multitask model. Specific details regarding the transformer model architecture and multitask model architecture can be found in SI Section 1.

An overview of our structure elucidation framework is shown in Figure 1. The top row of Figure 1 illustrates our full end-to-end multitask structure elucidation model, which takes in the ^1^H and/or ^13^C NMR spectra and predicts both the substructures in the molecule and the molecule’s structure. Our framework uses an end-to-end approach, without the explicit prediction of the substructures as an intermediate employed in our previous work,^20^ to avoid the significant loss of information when compressing the latent features of the model to the low-dimensional representation afforded by the substructure arrays. The red nodes denote the input layers to the model, the yellow nodes denote the hidden latent features generated within the model, the blue and white nodes represent the weights of the substructure elucidation and structure elucidation models, respectively, and the green nodes represent the outputs of the model. A precise schematic detailing the dimensions of the layers is provided in SIFigure 1. The bottom row of Figure 1 shows our approach to the substructure-to-structure problem, where molecular structures, represented by SMILES strings, are formed out of molecular fragments, represented by substructure arrays. As we will show below, pretraining on the task of structure elucidation from substructures substantially improves the accuracy of the full multitask structure elucidation model. Hence, we first demonstrate how a transformer can be trained on this task before showing how this can be integrated into the complete end-to-end multitask framework.

### Substructure-to-Structure Prediction

We first consider the problem of deducing a molecular structure from knowledge of the presence or absence of a set of substructures, which we define as small fragments of a molecule with defined bonding relationships. This task is inspired by the way chemists commonly interpret NMR spectra, using peaks to identify fragments and assembling those fragments into candidate structures. Manually converting a relatively small number of substructures into a molecular structure is already challenging even for an experienced chemist, and it quickly becomes impractical when confronted with dozens or hundreds of substructures, which is necessary for accommodating the complexity of the structure space. Hence, devising an automated strategy for structure elucidation from these molecular fragments can itself help accelerate structure determination.

Here, we treat the substructure-to-structure problem as a language translation problem, where we translate a sequence of substructures into a sequence of tokens that can be combined to form a molecule’s SMILES string, recovering the molecular connectivity. Our approach is outlined in the bottom row of Figure 1, where we use a transformer model architecture for this task. Autoregressive transformer models have been shown to excel at translation tasks owing to the strong inductive bias provided by the multihead attention mechanism, which captures global correlations and uses that information to generate the target sequence in a conditioned manner.^29^ This strength is critical in a chemical context because the overall connectivity of a molecule arises from considering all molecular fragments collectively, and many fragments may or may not overlap depending on the molecular context. Here, we adapt a full encoder-decoder transformer architecture for the substructure-to-structure task.

The inputs to the substructure-to-structure transformer are a binary vector with each entry corresponding to the presence or absence of a specific substructure and a start token. Using only this information, the transformer generates a SMILES string token by token, which it continues to build until it terminates upon prediction of the stop token.

To train the transformer on this task, we started with a set of ∼143 thousand molecules from the SpectraBase^30^ database containing only C, N, and O as the non-hydrogen atoms and combined it with a set of ∼3 million molecules randomly sampled from the GDB-17 data set^14^ to create a final data set of ∼3.1 million molecules. After canonicalizing all the SMILES strings using RDKit,^31^ the substructure vector for each molecule was computed using RDKit’s substructure match functionality against the set of 957 substructures used in our previous work^20^ represented as SMART strings. For the target that the transformer uses in decoding, the canonicalized SMILES were tokenized using a regular expression,^32^ and an alphabet was determined from all unique tokens across all SMILES to ensure that all SMILES can be represented during training and testing. Further details of our transformer training data set are provided in SI Section 2.1.

During training, the data set was split into a training, validation, and test set using a random split, with 80% of the data used for training, 10% used for validation, and 10% used for testing. To produce a more compact representation of the binary substructure arrays for the embedding, an integer array was created that lists the index (1 to 957) of only the present (nonzero) substructures for each molecule. Each unique SMILES token was also assigned to an integer index and used for embedding. Right padding was applied to ensure that all substructure vectors and SMILES vectors in a batch were of the same length before being passed into the transformer. The padding was masked during the training. Further details of our transformer training procedure are provided in SI Section 3.1.

**Figure 2:**
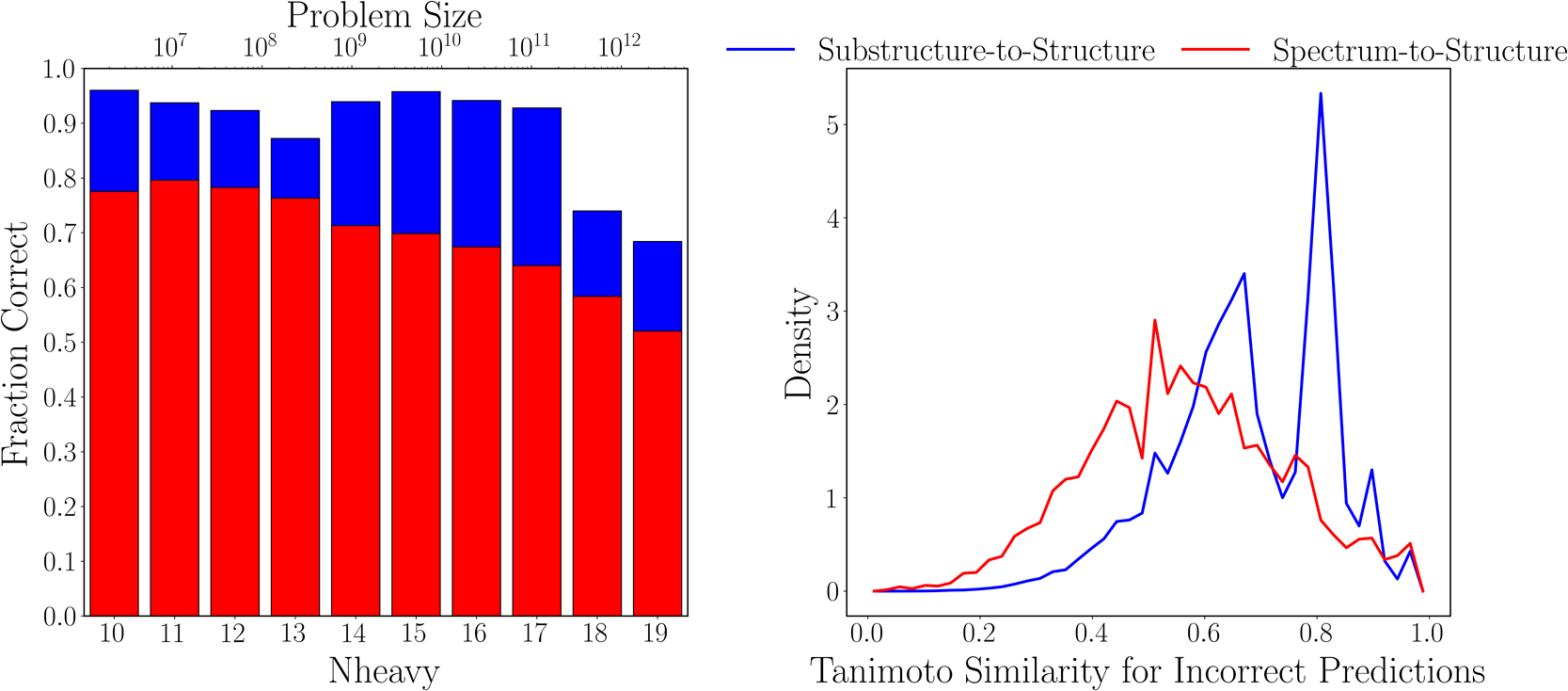
(Left) Transformer and the best multitask model test accuracy as a function of the problem size. The problem size is determined by extrapolating an exponential fit to the number of molecules in GDB-9,^33^ GDB-11,^34^ GDB-13,^35^ and GDB-17, and the plot begins with the number of possible structures for 10 heavy (non-hydrogen) atoms. (Right) Distribution of Tanimoto similarities of the best incorrect predictions relative to the target molecule after removing correct predictions and invalid SMILES.

**Figure 3:**
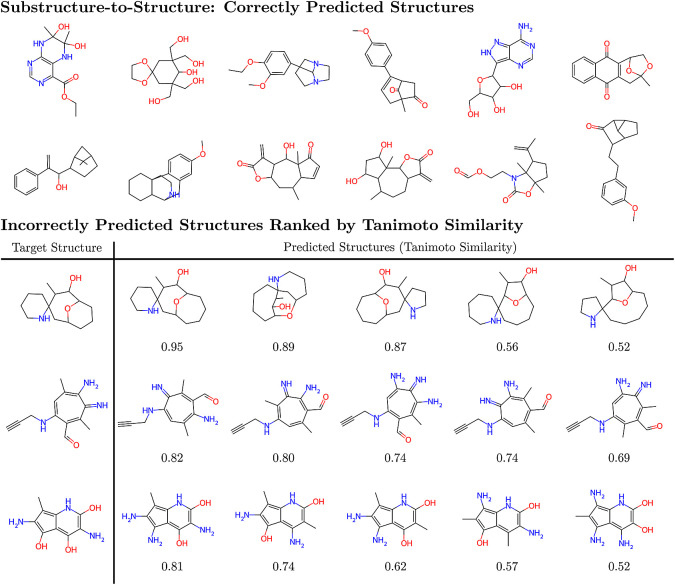
(Top) Examples of molecules that were correctly predicted by the transformer model. The molecules shown have between 23 to 65 substructures, and examples of correctly predicted molecules and their constituent substructures are shown in SI Figures 5–7. (Bottom) Examples of molecules with incorrect predictions from the transformer model. The number beneath each predicted molecule is the Tanimoto similarity between the prediction and target.

To test the trained transformer model for the substructure-to-structure task, top-*k* random sampling^36^ was used to generate SMILES from a given substructure array with *k* = 5, and 15 predictions were generated for each input. Using these 15 generated SMILES, the prediction was considered correct if one of these molecules, upon canonicalization, matched the exact canonical SMILES of the actual molecule. On the test set, the model achieved an accuracy of 93.2%. Examples of molecules correctly predicted by the transformer model are shown at the top section of Figure 3, demonstrating that the model can deduce complex molecular structures using only substructures as input. These example molecules require the transformer to assemble between 23 and 65 substructures into the correct molecule. The difficulty of this task is illustrated in SI Figures 5–7, which show molecules that were correctly predicted by the substructure-to-structure model and the substructures that were provided to it. The test set accuracy as a function of the number of heavy atoms is shown as blue data in the left panel of Figure 2 for up to 19 heavy atoms. Notably, the accuracy of the substructure-to-structure model shows little variation as the problem size grows. For example, at 10 heavy atoms, where the problem size is ∼2 million, the accuracy is 96.0% and only drops to 92.8% by 17 heavy atoms, where it has increased to ∼200 billion. However, the accuracy drops below 80% for the molecules with 18 or 19 heavy atoms. Although this sudden decrease could be explained in part by the problem size entering the trillions for molecules of that size, a more likely explanation might be that GDB-17 only contains molecules with up to 17 heavy atoms, while the SpectraBase data set, which does contain molecules with 18 and 19 heavy atoms, is much smaller. Hence, whereas 59% in our training data come from molecules with 17 heavy atoms, only 1% come from molecules with 18 or 19 heavy atoms, meaning that the model has had fewer opportunities to learn how to predict systems of that size. However, the fact that the model retains reasonable accuracy for these larger molecules suggests that the approach is generalizable. Since computing the substructure vectors is facile using RDKit, the set of substructures could also easily be expanded to cover fragments more commonly encountered in larger molecules, which would be expected to further improve performance.

To better understand the failure modes of the substructure-to-structure model, we examine the distribution of Tanimoto similarities^37^ computed between the model’s best incorrect prediction and the correct molecule in cases where the correct molecule was not predicted. This is shown for the test set as the blue line in the right panel of Figure 2. Overall, 89.6% of the best incorrect predictions have a similarity to the correct molecule that is greater than or equal to 0.50, with an average similarity of 0.68. To see how these similarity scores correlate to the molecular structure of the incorrect predictions, the bottom section of Figure 3 shows some example target molecules and incorrect predictions at varying degrees of Tanimoto similarity. We see that even for incorrect predictions at similarities near 0.50, the predictions already contain many of the desired functional groups and bonding motifs of the target, albeit with incorrect connectivity. As the similarity increases, the connectivity and structure of the predictions improve, and at similarities near 0.80 and above, the incorrect predictions are very close to the target, often differing by a few atoms or bonds. This is encouraging, as it shows that the transformer is capable of learning how to map the binary substructure representation to real molecular fragments, leading to the recovery of a significant portion of the target molecule scaffold even in cases where the exact correct molecule is not predicted. Considering both the high proportion of best incorrect predictions above 0.50 and the high mean similarity, these results show that even when the model returns incorrect predictions, the molecules generated can still provide meaningful structural information about the target molecule.

### Spectrum-to-Structure and Spectrum-to-Substructure Prediction Using a Multitask Workflow

We now focus on the more challenging problem of directly elucidating the molecular structure and substructures from the ^1^H and ^13^C NMR spectra, which we refer to as spectrum-to-structure and spectrum-to-substructure prediction, respectively. Our goal is to design a model capable of predicting a molecule’s structure and the substructures it contains using only the spectral data as inputs, operating in an end-to-end fashion i.e. taking the spectrum and directly predicting the structure it corresponds to and the substructures within that molecule with no intermediate steps. The workflow for our approach is shown in the top row of Figure 1. As we will emphasize further below, an important component of training this multitask spectrum-to-structure model is the initialization of the transformer using the weights obtained from training on the substructure-to-structure task (as indicated by the arrow in Figure 1 from the bottom workflow to the multitask model on the top).

For training and testing the spectrum-to-structure model, we used ^1^H and/or ^13^C NMR spectra for the ∼143 thousand set of molecules from SpectraBase predicted using MestreNova,^38^ using the same train-validation-test split of the molecules as on the substructure-to-structure task. To effectively utilize all portions of the spectral signal, we took a minimal preprocessing approach to the spectra to retain as much information as possible. Specifically, since the ^1^H NMR contains detailed splittings and rapidly varying features, we processed the spectrum by mildly reducing the dimensionality of the data by linearly interpolating the spectrum of 32768 values down to a grid of 28000 values corresponding to a shift range of −2 to 12 ppm with a resolution of 0.0005 ppm. To assist the numerical stability when fitting the multitask model while retaining the relative intensities of all peaks, all intensities were normalized to between 0 and 1 by dividing each spectrum by the intensity of its highest peak. Normalization did not have a statistically significant impact on the model accuracy (see SI Section 4). For the ^13^C NMR spectra, because the intensities are not generally reliable indicators of the relative number of nuclei and the peak shapes are not informative because peaks are typically decoupled, we processed the spectra by extracting the chemical shifts and binning them into 80 bins spanning the shift range of 3.42 to 231.3 ppm. Further details of our multitask model training data set are provided in SI Section 2.2.

During training, we passed the processed ^1^H NMR spectra through a one-dimensional convolutional neural network (CNN) to downsample the signal into a lower-dimensional space. For the processed ^13^C NMR spectra, since it is a binary vector, we embedded it into a dense vector representation. If both spectra were being used as input to the spectrum-to-structure model, then their features were concatenated. If only one spectrum was being used as input, then only the features for that spectrum were retained. These features were then passed through a full encoder-decoder transformer for structure elucidation, which outputs SMILES strings, and a transformer encoder for substructure elucidation, which outputs substructure probability arrays as shown schematically on the top row of Figure 1. Further details of our multitask model training procedure are provided in SI Section 3.2.

Table 1 shows the structure elucidation accuracy on the test set as a function of both the spectral data used (^1^H and/or ^13^C NMR) and if weights from a transformer pretrained on the substructure-to-structure task were used; in other cases, randomly initialized weights were used. Structure elucidation accuracy was tested in the same way as for the substructure-to-structure task, where a target was considered correctly predicted if its canonical SMILES string appeared within the set of 15 predictions upon canonicalization. The highest accuracy of 69.6% (Table 1, third row) is obtained when using both the ^1^H and ^13^C NMR spectra with a pretrained transformer for structure elucidation. Without using the pretrained transformer, the structure elucidation accuracy drops significantly from 69.6% to 53.3%, demonstrating the importance of the pretraining step to the success of the overall multitask framework. This result suggests that pretraining on the substructure-to-structure task helps the model learn a chemically relevant latent space that translates well when integrated into the multitask framework.

**Table 1:** Test Set Structure Elucidation Accuracy and Substructure Elucidation *F*_1_ Score of the Multitask Model As a Function of the Type of Data Used and Weight Initialization for the Transformer Component

Data Used	Pretrained Transformer	Structure Accuracy (%)	Substructure *F*_1_ Score
^13^C NMR Only	Yes	22.0	0.69
^1^H NMR Only	Yes	59.6	0.81
^1^H + ^13^C NMR	Yes	69.6	0.86
^1^H + ^13^C NMR	No	53.3	0.86

To investigate the relative importance of ^1^H and ^13^C NMR as inputs, we trained two versions of the model using either ^1^H or ^13^C NMR as the sole input. The accuracy of the model is significantly better with only ^1^H NMR (59.6%) than with only ^13^C NMR (22.0%), reflecting the fact that ^1^H NMR contains far more information about the molecule’s connectivity relative to ^13^C NMR, thus playing a more critical role in structure elucidation. However, the best accuracy is obtained only when combining both spectra, showing that they are complementary to each other in terms of the information they each provide. We also note that using ^1^H NMR alone with the pretrained transformer gives a greater structure prediction accuracy than that obtained using both ^1^H and ^13^C NMR data without the pretrained transformer, highlighting further the advantages of this approach.

**Figure 4:**
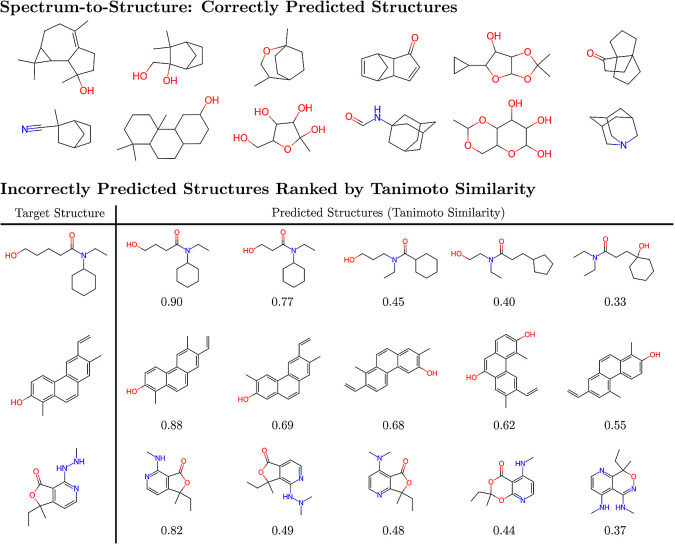
(Top) Examples of molecules that were correctly predicted using the multitask spectrum-to-structure model. (Bottom) Examples of molecules with incorrect predictions from the multitask model. The number beneath each predicted molecule is the Tanimoto similarity between the prediction and target.

The left panel of Figure 2 shows in red the structure elucidation accuracy of the spectrum-to-structure multitask model as a function of the number of heavy atoms. In comparison to the substructure-to-structure model, there is a more systematic decrease in accuracy with the size of the molecule, decreasing from 77.5% at 10 heavy atoms to 52.0% at 19 heavy atoms. However, this decrease in accuracy is dwarfed by the increase in the problem size that grows combinatorially and thus increases over 5 orders of magnitude over that range of molecule sizes. Hence, not only does the model’s accuracy decay extremely slowly compared to the growth of the number of possible structures, it is able to achieve this impressive scaling without any reliance on the molecular formula, molecular weight, or other information about the system that would otherwise constrain the problem. The top section of Figure 4 shows some of the molecules that were correctly predicted, emphasizing that the multitask model can elucidate the structures of molecules solely from their ^1^H and ^13^C NMR spectra across a wide range of chemical motifs that would be extremely challenging without additional information. These correctly predicted molecules were in each case one of the 15 predictions generated by the model. SI Figures 8 and 9 illustrate the other predictions made for two of these molecules, demonstrating that even the incorrect predictions are structurally similar to the correct prediction and therefore are also a useful starting point for further analysis.

For the molecules where the spectrum-to-structure model was unable to predict the correct molecule, we can again use Tanimoto similarities to assess how close the predicted molecule was. The distribution of Tanimoto similarities of the best incorrect predictions for the spectrum-to-structure task is colored red in the right panel of Figure 2. From this we see that compared to the substructure-to-structure task, the distribution of similarity scores is shifted to lower values, with only 64.7% of incorrect predictions having a similarity of 0.50 or greater with a mean similarity of 0.56. The lower similarity scores relative to the substructure-to-structure task reflect the fact that direct structure elucidation from only the NMR spectra is a considerably more challenging inverse problem that is less constrained than constructing the structure from a set of substructures. However, the bottom section of Figure 4 shows that the molecules with the highest Tanimoto similarity obtained from incorrect predictions of the multitask model still contain many of the chemical motifs expected in the target compound and so still provide insight into the system that is valuable when deducing the molecular connectivity.

The multitask model also outputs a prediction for the target molecule’s substructure profile, which can be interpreted as the probability of a given molecular fragment being present in the molecule based on its NMR spectra. These profiles, which provide additional information as to which of the 957 substructures are present in the system, are useful in cases where the model does not arrive at the correct structure, since they could be used by a chemist to infer other possible structures of the molecule. To evaluate the substructure elucidation accuracy, one must be careful to take into account that most molecules only contain a relatively small fraction of the total 957 substructures, and therefore, the profiles are dominated by zeros (which indicate the absence of a substructure). This leads to a highly imbalanced classification problem, where it is insufficient to only use accuracy as a performance metric. To address this issue, Table 1 shows the *F*_1_ score that balances the need to account for true and false positives as well as negatives. From this, we see that for this spectrum-to-substructure task, while the performance improvements in the *F*_1_ score of using both ^1^H and ^13^C NMR spectra over either individually are retained, the pretraining of the transformer has a negligible impact on the substructure elucidation. This is perhaps expected since although the multitask model produces both a prediction of structure and substructures in an end-to-end fashion, the pretrained transformer weights arise from the task of substructure-to-structure prediction, which is a downstream task from substructure prediction.

**Figure 5:**
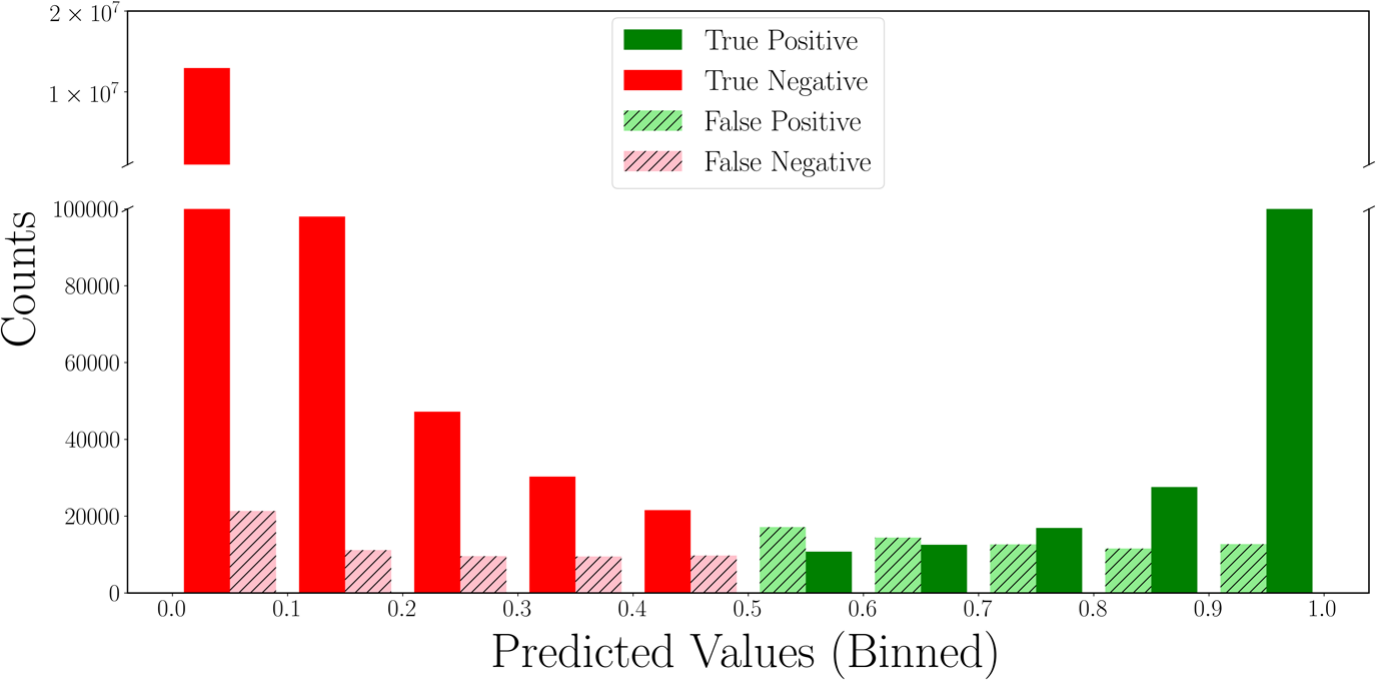
Distribution of true/false positives and true/false negatives as a function of the probability predicted by the multitask model using both ^1^H and ^13^C NMR as inputs and a pretrained transformer. A decision boundary of 0.5 is used to distinguish between positives and negatives.

To quantify the model’s predictive performance on the spectrum-to-substructure task beyond measures like the *F*_1_ score, we can examine the distribution of the model’s predicted probabilities. Figure 5 shows the distribution of the model predictions on the test set. In cases where the model predicts a low probability of a substructure being present (<0.1), the prediction is 99.8% accurate. Conversely, when the model predicts a high probability of a substructure being present (>0.9), the prediction is 96.3% accurate. The slightly lower accuracy in the case of predicting positive substructures arises from the significant imbalance of positives to negatives since in the training data each molecule only contains a small fraction of the 957 substructures, and so there are far more true negatives than true positives in the data set. The uncertainty of the model increases when moving away from the extremes of the predicted probability toward the decision boundary of 0.5 between positive and negative predictions; however, only 2.6% of substructure predictions have a probability within the range of 0.1 < *p* < 0.9, so the predicted probability from the model is a strong indicator of the presence or absence of a substructure the majority of the time.

## Conclusion

In summary, we have introduced a multitask machine learning approach for direct structure and substructure elucidation from ^1^H and ^13^C NMR spectra that leverages pretraining an encoder-decoder transformer on the related task of substructure-to-structure elucidation. By integrating this transformer architecture into our multitask framework, we have shown that our end-to-end model is able to predict the structure correctly 69.6% of the time within the first 15 predictions using only the ^1^H and ^13^C NMR as input for systems of up to 19 heavy atoms. Furthermore, our end-to-end model can simultaneously predict which substructures are present in a molecule with an accuracy of 96.3% when the predicted probability is above 0.9 and which substructures are not present with 99.8% accuracy when the predicted probability is below 0.1, with only 2.6% of the predicted substructure probabilities not falling within those ranges. What is particularly remarkable is that while the problem size (number of possible molecules that can be constructed consistent with basic chemical bonding rules) grows combinatorially with the number of heavy atoms from 10 to 19 heavy atoms our multitask model shows only a 25.5% decrease in accuracy over a range in which the number of possible molecules increases by 5 orders of magnitude. This suggests that our approach to the inverse problem of spectrum-to-structure is scalable to larger chemical systems. Our model thus provides an efficient avenue for direct spectrum-to-structure elucidation from ^1^H and ^13^C NMR spectra without dependence on any prior chemical information, such as the molecular formula or molecular fragments.

This work sets the stage for future developments of this multitask framework to elucidate even larger molecules with an extended range of elements and the prediction of stereochemistry. Enabling these developments will require expanding the set of substructures used to pretrain the transformer with substructures containing additional elements and larger, more complicated chemical motifs, such as larger ring systems or protecting groups. Relative stereochemistry could be incorporated within our existing multitask framework by training on SMILES representations containing the characters specifying stereocenters and *cis-* and *trans-*double bond configurations and the corresponding NMR spectra of molecules. This could be achieved by identifying all of the stereocenters in the current training set, enumerating all possible stereoisomers, and using these to augment the training set. An alternative approach to tackling the stereochemistry problem is to predict composition and connectivity as shown here and then do forward prediction of the spectra of different stereoisomers of candidate structures and compare them to the input spectra to identify the most likely stereoisomer.

Although in this work we have concentrated on absolute structure prediction with no chemical knowledge of the compound beyond its NMR spectra, this framework could also be easily adapted for cases where some information about the chemical system is available, such as in the case of reaction prediction or retrosynthesis, where the starting materials of a reaction are known, and hence, the problem is considerably more constrained. We also envision this technology to be used in a complementary manner with other existing structure elucidation, reaction prediction, or retrosynthesis frameworks as a way to rapidly detect structural or substructural changes in a reaction pathway or as a way to quickly constrain the number of possible candidate structures to a point where more refined techniques can be applied tractably. To enable such adaptations and future developments, we have released the code on GitHub^39^ and provided the training, validation, and test data on Zenodo.^40^ Our approach opens up new possibilities in the field of ML-driven structure elucidation by introducing a fast and efficient structure elucidation framework that can operate in an unsupervised manner without relying on additional knowledge. The trained model, which is provided in our GitHub repository,^39^ can make a full prediction of the structure and substructures of an input ^1^H and ^13^C NMR spectra for a system of 19 heavy atoms in under 3 s even on standard CPU hardware (a single core of an AMD Ryzen 7 3700X 8-core processor). This framework thus has the potential to provide a highly accessible technology to greatly accelerate characterization and chemical discovery at levels ranging from high school chemical education to industrial research settings. Acquiring large data sets of high-quality experimental NMR spectra in the form needed to train our model (i.e., original FID files) currently poses a challenge but is essential to creating a model that encodes the nuances of real spectra that are not fully captured by the simulated spectra used to train the current model. For example, the accuracy of the framework drops from 69.6% to 33.0% when used to predict the structures from 106 experimental ^1^H and ^13^C NMR spectra of molecules employed in our previous work^20^ (see SI Section 5), emphasizing the potential accuracy improvements that could be achieved on experimental spectra if more training data was available. Ultimately, we envision a community-driven effort to aid in curating a large database of experimental NMR spectra, which will help the model learn to predict the structure from spectra collected under a variety of conditions. This synergistic effort to assist community chemical characterization efforts in tandem with improving the prediction model provides an opportunity to greatly improve the accuracy of this tool while supporting the chemistry community.

## Supporting Information Available

Architectures and hyperparameters for all models presented in the main text, details on the data curation for the substructure-to-structure, spectrum-to-structure, and spectrum-to-substructure tasks, optimization protocol and learning curves for all models presented in the main text, and additional examples of correctly predicted molecules with the associated spectra or substructures (PDF)
