# Supplementary material for: Accurate and Efficient Structure Elucidation from Routine One-Dimensional NMR Spectra Using Multitask Machine Learning
Source: ACS Cent Sci. 2024 Nov 13;10(11):2162–70. doi: 10.1021/acscentsci.4c01132 (PMC11613330; doi:10.1021/acscentsci.4c01132)
Supplement: Supplementary file 1 — oc4c01132_si_001.pdf [file oc4c01132_si_001.pdf]

# Supplemental Information: Accurate and efficient structure elucidation from routine one-dimensional NMR spectra using multitask machine learning

Frank Hu,[†] Michael S. Chen,[‡] Grant M. Rotskoff,[*,†] Matthew W. Kanan,[*,†] and Thomas E. Markland[*,†]

†*Department of Chemistry, Stanford University, Stanford, California 94305, United States*

‡*Simons Center for Computational Physical Chemistry, Department of Chemistry, New York University, New York, New York 10003, United States*

E-mail: rotskoff@stanford.edu; mkanan@stanford.edu; tmarkland@stanford.edu

## Contents

**1 Model architectures** **3**

**2 Data curation** **5**

2.1 Substructure-to-structure transformer . . . . . . . . . . . . . . . . . . . . . 5

2.2 Spectrum-to-structure and spectrum-to-substructure multitask model . . . . 6

**3 Optimization protocol** **6**

3.1 Substructure-to-structure transformer . . . . . . . . . . . . . . . . . . . . . 6

3.2 Spectrum-to-structure and spectrum-to-substructure multitask model . . . . 8

4 Effect of spectrum normalization on model performance 11

5 Early stopping on experimental data 12

References 23

# 1 Model architectures

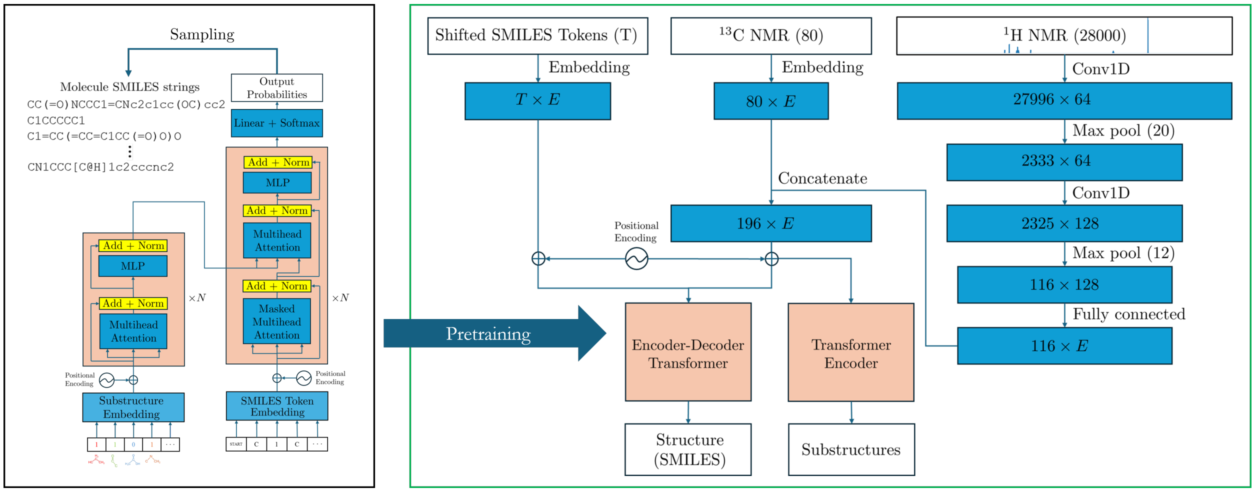


Figure 1: Diagram of the multitask workflow during training, with the substructure-to-structure transformer (left, black box) feeding into the overall multitask architecture (right, green box). $E$ refers to the model embedding dimension, $T$ is the length of the target SMILES string in tokens, and $N$ is the number of encoder/decoder layers in the transformer. The transformer (left, black box) uses the PyTorch Transformer class. Conv1D is a one-dimensional convolution using the PyTorch Conv1d layer, MLP (a multi-layer perceptron) refers to a feed-forward neural network as described in the original transformer paper,[1] max pooling is performed using the PyTorch MaxPool1d layer, embedding is done using the PyTorch Embedding layer, and Norm refers to layer normalization.

For the substructure-to-structure transformer (Figure 1, black box), we use an encoder-decoder transformer[1] as implemented in PyTorch.[2] Table 1 details the exact architecture of the final model used for the results in the main text. All models used absolute positional encoding based on sinusoidal functions of different frequencies[1] as described by the following equations:

$$PE_{(pos,2i)} = \sin(pos/10000^{2i/d_{model}})$$

$$PE_{(pos,2i+1)} = \cos(pos/10000^{2i/d_{model}})$$

where $pos$ is the position, $i$ is the dimension, and $d_{model}$ is the model embedding dimension. Positional encodings are added to the embeddings for both the substructures and the SMILES tokens.

Table 1: Substructure-to-structure architectural parameters, descriptions, and their values.

| Parameter | Description | Value |
|---|---|---|
| d_model | Embedding dimension of the model | 128 |
| dim_feedforward | Hidden layer dimension of the feed forward neural network within the transformer blocks | 1024 |
| source_size | Total number of possible token values for embedding substructure sequences | 958 |
| src_pad_token | The index used for padding source sequences to the same length | 0 |
| target_size | Total number of possible token values for embedding SMILES token sequences | 24 |
| tgt_pad_token | The index used for padding target sequences to the same length | 21 |
| num_encoder_layers | The number of encoder layers | 6 |
| num_decoder_layers | The number of decoder layers | 6 |
| nhead | The number of heads used in multihead attention | 8 |
| activation | The activation function used for intermediate encoder/decoder layers | relu |
| dropout[3] | The probability for a particular element of an input tensor to be randomly set to 0 | 0.1 |
| layer_norm_eps | The constant used for numerical stability in layer normalization[4] | 1E-5 |

Table 2: Spectrum-to-substructure encoder architectural parameters, descriptions, and their values.

| Parameter | Description | Value |
|---|---|---|
| d_model | Embedding dimension of the model | 128 |
| dim_feedforward | Hidden layer dimension of the feed forward neural network within the transformer blocks | 1024 |
| num_encoder_layers | The number of encoder layers | 4 |
| nhead | The number of heads used in multihead attention | 4 |
| activation | The activation function used for intermediate encoder/decoder layers | relu |
| dropout[3] | The probability for a particular element of an input tensor to be randomly set to 0 | 0.1 |
| layer_norm_eps | The constant used for numerical stability in layer normalization[4] | 1E-5 |

For the spectrum-to-structure and spectrum-to-substructure multitask model, the architecture of the convolutional embedding for the $^1$H NMR and embedding for the $^{13}$C NMR is shown on the right side in the green box of Figure 1. The architecture of the encoder-decoder transformer component is the same as in Table 1, and Table 2 describes the architecture of the encoder component used for substructure elucidation in the final multitask model. An important step in obtaining the substructure profile from the encoder is aggregating the information from the higher-dimensional raw encoder output into the lower-dimensional format of the substructure profiles. To this end, we adapt a method called sequence pooling.[5] Given an input sequence $\mathbf{x}_0$ and a function $f$ parameterized by a neural network, sequence

pooling performs the following operations in order:

$$\mathbf{x}_L = f(\mathbf{x}_0) \in \mathbb{R}^{N \times T \times E} \tag{1}$$

$$\mathbf{x}'_L = \text{softmax}(g(\mathbf{x}_L)^T) \in \mathbb{R}^{N \times 1 \times T} \tag{2}$$

$$\mathbf{z} = \text{squeeze}(\mathbf{x}'_L \mathbf{x}_L) \in \mathbb{R}^{N \times E} \tag{3}$$

where $N$ is the batch size, $T$ is the sequence length, $E$ is the embedding dimension, and $g(\cdot) \in \mathbb{R}^{E \times 1}$ is a learnable linear transformation. This can be understood as attending across the sequence dimension of the data after processing it through the encoder and assigning importance weights to each element in the sequence before aggregation.

# 2 Data curation

## 2.1 Substructure-to-structure transformer

To generate the data for training the substructure-to-structure transformer model, we started with a set of 142894 SMILES strings containing only C, N, O, and H atoms from the SpectraBase[6] dataset. We canonicalized all the SMILES strings using RDKit[7] and removed all stereochemical information from the strings, including designation of double bond stereochemistry. We combined this set of 142894 SMILES with randomly sampled SMILES from the GDB-17 dataset[8] to create a final dataset of 3116791 SMILES strings. Using the set of 957 substructures from our previous work,[9] we constructed the substructure arrays for this dataset by performing a substructure search, generating a binary vector of length 957 for each molecule where a “1” means a substructure is present and a “0” means a substructure is absent. SMILES strings were tokenized using a regular expression.[10]

## 2.2 Spectrum-to-structure and spectrum-to-substructure multitask model

To generate data for training the spectrum-to-structure and spectrum-to-substructure multitask model, we converted the set of 142894 SMILES strings from SpectraBase into two-dimensional mol files using open babel[11] and then computed the $^{1}$H and $^{13}$C NMR spectra using MestreNova[12] version 14.2.

$^{1}$H NMR spectra were computed with a line width of 0.75 Hz using deuterated chloroform as the solvent. Labile protons were excluded from the calculation of the spectra. The spectra were output as grids of 32768 intensities which were then interpolated down to grids of 28000 values spanning a ppm shift range of -2 to 12 ppm with a resolution of 0.0005 ppm. Each spectrum was normalized by dividing by the intensity of its highest peak, thereby normalizing all intensities to between 0 and 1 to assist with model stability. $^{13}$C NMR spectra were computed with a line width of 1.50 Hz in proton decoupled mode. Chemical shifts were extracted from the spectrum and binned into 80 bins spanning the shift range of 3.42 to 231.3 ppm.

# 3 Optimization protocol

## 3.1 Substructure-to-structure transformer

The substructure-to-structure transformer model was trained using a batch size of 32 with a constant learning rate of $1 \times 10^{-5}$ and a weight decay of $1 \times 10^{-5}$ using the ADAM[13] optimizer implemented in PyTorch. The loss function for the substructure-to-structure model was a cross entropy loss between the model's output probability distribution and the correct sequence of tokens for the target SMILES string. The dataset composed of the 3116791 SMILES strings and substructure arrays was partitioned using a random splitting with 80% used for training, 10% used for validation, and 10% used for testing. Early stopping was

used to prevent overfitting by monitoring the loss on the validation set. The model was optimized for 312 epochs and the final model selected was that with the lowest validation loss. This model was the one used to compute the results shown in the main text. The final substructure-to-structure model was trained for 308 epochs, at which point the minimum validation loss was attained. The learning curve for the training of the substructure-to-structure model is shown in Fig. 2.

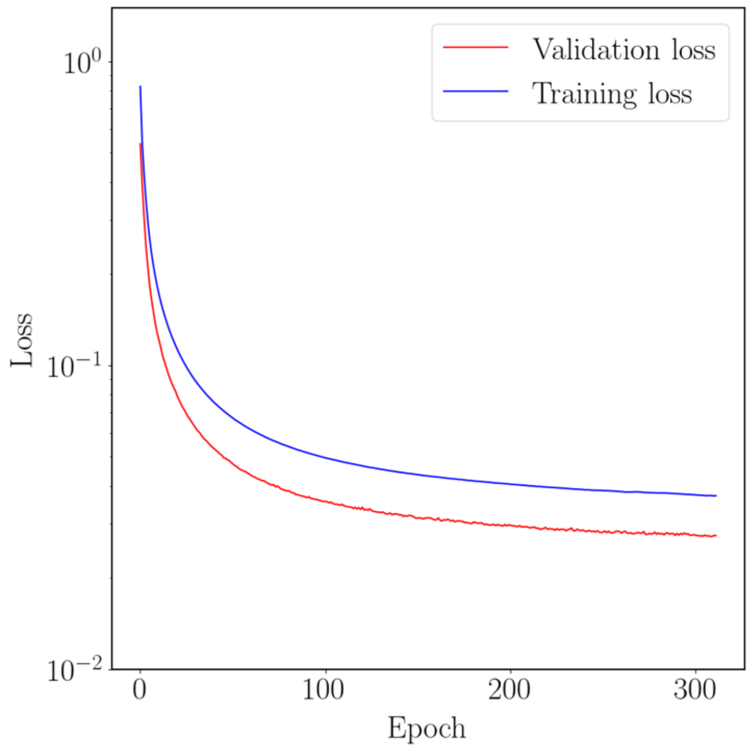


Figure 2: Training and validation loss curves for the transformer model used in the main text.

The reason that the validation loss is consistently lower than the training loss is because of the use of dropout[3] as a regularization technique when training the transformer model. During forward passes through the model when in the training stage, dropout randomly samples certain elements of the input tensor passed through the layer and sets the elements to zero with some probability $p$. This means that only a subset of the network's neurons are used when computing the training loss in each forward pass. During validation, dropout layers are disabled and all neurons within the model are used (and all neurons are also used

when evaluating the test set with the final fit model). Thus, the training loss is computed using a subset of the model's neurons whereas the validation loss is computed over the full model, leading to a lower validation loss than training loss. To demonstrate that the expected behavior (train loss less than or equal to validation loss) is obtained when not using dropout layers we have re-evaluated the full model's (i.e. with all neurons used) loss on the train and validation set from 10 saved checkpoints and show that in each case, the validation loss is higher than the training loss. These results are shown in Table 3. Furthermore, by enabling dropout layers during validation and refitting the transformer model, the model's validation loss is consistently higher than the training loss over the first 100 epochs, as expected. This is shown in Figure 3 for two different train-validation-test splittings of the 3M dataset.

Table 3: Training and validation loss from checkpoints re-evaluated on the training set with all neurons. The validation loss is observed to be consistently higher than the training loss when all neurons of the model are used to evaluate the training set.

| **Epoch** | **Validation Loss** | **Training loss** | **Validation - Training** |
|---|---|---|---|
| 301 | 0.02750 | 0.02300 | +0.00449 |
| 302 | 0.02747 | 0.02295 | +0.00452 |
| 303 | 0.02743 | 0.02289 | +0.00454 |
| 304 | 0.02739 | 0.02294 | +0.00445 |
| 306 | 0.02739 | 0.02270 | +0.00468 |
| 307 | 0.02748 | 0.02296 | +0.00452 |
| 308 | 0.02729 | 0.02269 | +0.00460 |
| 309 | 0.02735 | 0.02273 | +0.00463 |
| 310 | 0.02751 | 0.02278 | +0.00473 |
| 311 | 0.02750 | 0.02291 | +0.00459 |

## 3.2 Spectrum-to-structure and spectrum-to-substructure multitask model

The four spectrum-to-structure and spectrum-to-substructure multitask models, which correspond to different conditions of input data and whether a pretrained transformer was used, shown in Table 1 of the main text were trained using the following procedure. The models were trained using a constant learning rate of $1 \times 10^{-5}$ and a batch size of 64 without any

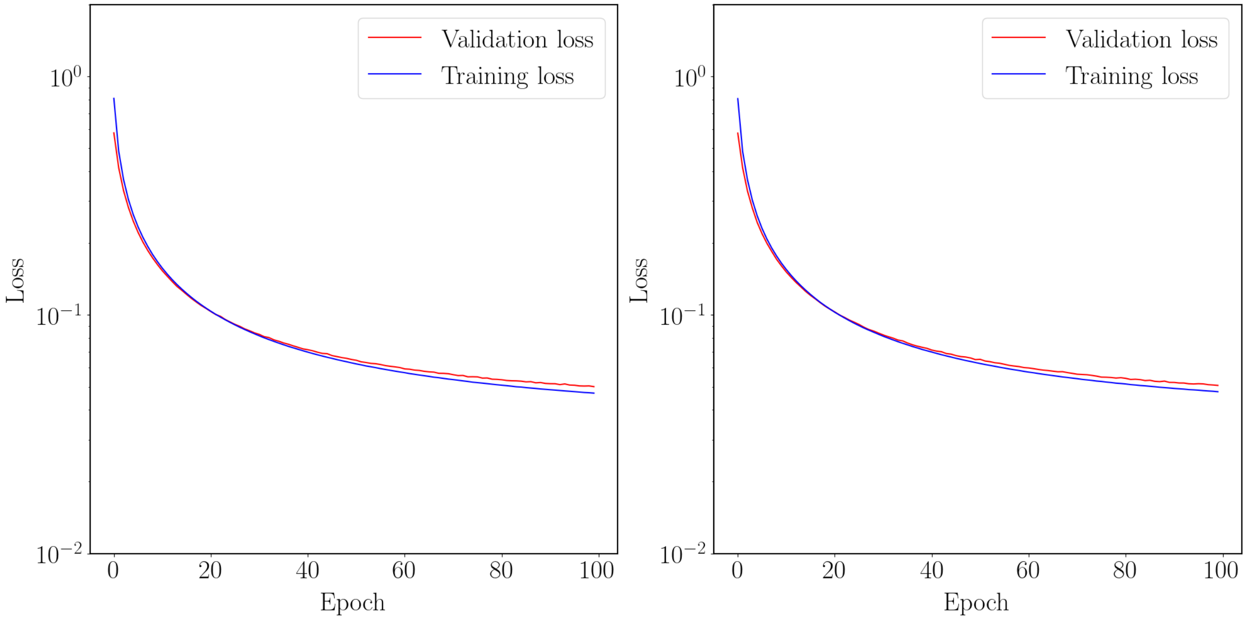


Figure 3: Training and validation loss curves over the first 100 epochs for two transformer models trained with a different splitting of the data and dropout enabled during validation. We see that in each case, the validation loss closely tracks the training loss and exceeds the training loss by the end of 100 epochs.

weight decay. The loss function being optimized for each multitask model is a weighted sum of the cross entropy loss for the SMILES prediction and a binary cross entropy loss for the substructure prediction. Given a prediction-target pair for the SMILES prediction $(\boldsymbol{y}_{smi}, \hat{\boldsymbol{y}}_{smi})$ and a prediction-target pair for the substructure prediction $(\boldsymbol{y}_{sub}, \hat{\boldsymbol{y}}_{sub})$, the total loss is computed as:

$$\mathcal{L}_{tot} = \alpha \cdot \mathcal{L}_{CE}(\boldsymbol{y}_{smi}, \hat{\boldsymbol{y}}_{smi}) + \beta \cdot \mathcal{L}_{BCE}(\boldsymbol{y}_{sub}, \hat{\boldsymbol{y}}_{sub})$$

where $\alpha$ and $\beta$ were set to $\alpha = \beta = 1$.

The dataset of 142894 SMILES strings, substructure arrays, and spectra was partitioned using the same train-validation-test split as in the substructure-to-structure task. For each of the four models early stopping was used to prevent overfitting by monitoring the loss on the validation set. The total number of epochs that each of the four models was trained for is shown in Table 4. The final model selected for each set of conditions was that with the lowest

validation loss with the number of epochs at which that loss was reached shown in Table 4 for each of the four models. The learning curves for the training of the spectrum-to-structure and spectrum-to-substructure multitask models are shown in Fig. 4. By comparing the learning curves we note that using a pretrained transformer not only improves the structure elucidation accuracy, as described in the main text, but also accelerates convergence relative to training the multitask model from scratch.

Table 4: Number of epochs each multitask model was trained to attain the minimum validation loss.

| **Data used** | **Pretrained Transformer** | **Total number of epochs trained** | **Final model epoch** |
|---|---|---|---|
| $^{13}$C NMR Only | Yes | 779 | 261 |
| $^{1}$H NMR Only | Yes | 521 | 288 |
| $^{1}$H + $^{13}$C NMR | Yes | 414 | 250 |
| $^{1}$H + $^{13}$C NMR | No | 465 | 456 |

# 4 Effect of spectrum normalization on model performance

Part of the data processing for the $^{1}$H NMR spectra described in the main text is normalization of the intensities of each spectrum such that the maximum intensity is 1 and the minimum intensity is 0. This feature normalization was done to assist with the numerical stability when training the model. To investigate if this normalization has any effect on the multitask model's performance, we trained 5 versions of the multitask models ($^{1}$H NMR + $^{13}$C NMR with the pretrained 3M transformer) with normalization of the $^{1}$H NMR spectrum and 5 versions of the multitask model without normalization. All models used the same dataset of 142894 molecules with the same training-validation-test split, with the only variation being the random seed used for initialization of the model. Table 5 shows the structure elucidation accuracy for all 10 training runs. The normalization had no impact on the substructure $F_1$ score, with each model obtaining an $F_1$ score of 0.86. Averaging across the 5 runs for each condition, we get an average accuracy of 70.2±0.84% with normalization and 71.4±0.90% without normalization. Thus, the average accuracy without normalization is within 1.2% of the average accuracy with normalization which is within statistical fluctiations (i.e., 1.5 standard deviations) of the models arising from different initializations.

Table 5: Structure accuracy of multitask models trained with and without normalization of the $^{1}$H NMR.

| **Structure Accuracy (%) With Normalization** | **Structure Accuracy (%) Without Normalization** |
|---|---|
| 69.6 | 72.1 |
| 69.2 | 70.1 |
| 69.8 | 72.7 |
| 70.8 | 71.0 |
| 71.5 | 71.2 |

# 5 Early stopping on experimental data

As a test of the multitask model's performance on experimental data, we used a set of 310 experimental spectra from our previous work[9] to perform validation and testing of a multitask model trained on simulated data. The experimental $^1$H NMR spectra were processed the same way as described in the main text while the experimental $^{13}$C NMR was binned using only 40 bins instead of 80 bins. To ensure consistency, we trained a new model using a reprocessed version of the training and validation set where the $^{13}$C NMR had only 40 bins instead of 80.

We trained a multitask model ($^1$H NMR + $^{13}$C NMR with the pretrained 3M transformer) using simulated data. Experimental data was used for the validation and test set with 214 spectra in the validation set and 106 in the test set. During training, the validation loss on the experimental data was monitored and used to perform early stopping to mitigate overfitting. The learning curve is shown in Figure 10. The model overfits very quickly to the simulated data, with the validation loss on the experimental data rapidly increasing after the first 30 epochs. The lowest validation loss was obtained at epoch 27, with a structure elucidation accuracy of 33.0% and a substructure $F_1$ score of 0.60. While this is lower than the numbers reported in the main text on the simulated data (69.6% and 0.86, respectively), this emphasizes that significant improvements might be attainable if sufficient quantities of experimental data were used to train the model instead of only simulated data.

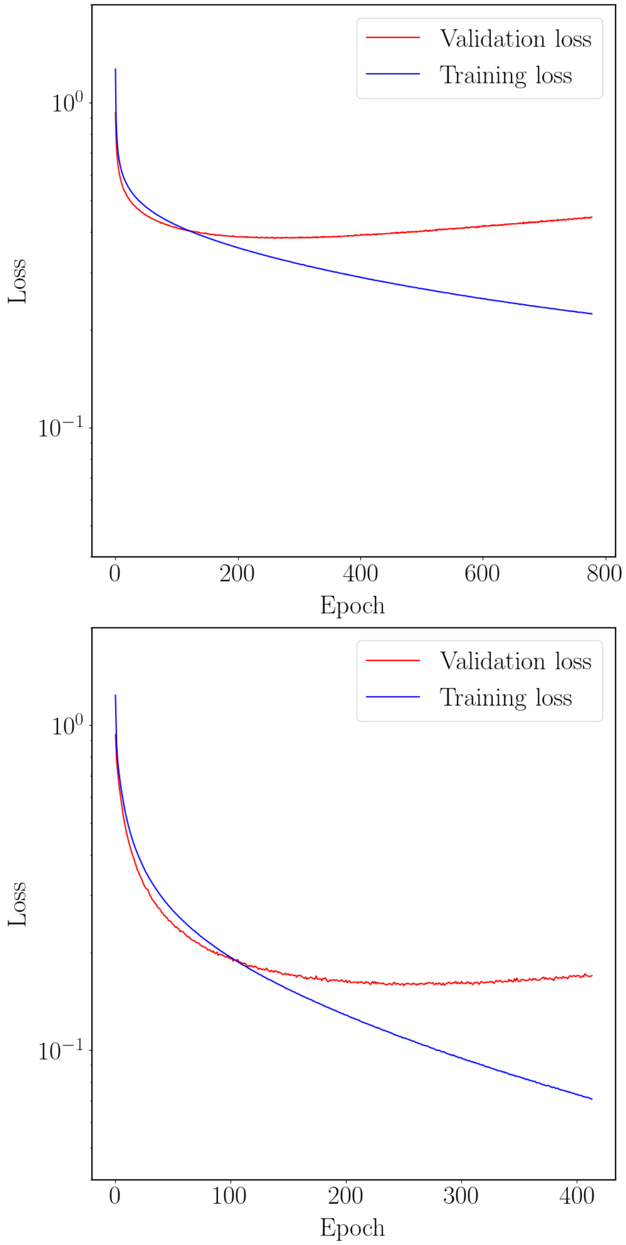


Figure 4: Training and validation loss curves for the multitask models used in the main text: (top left) $^{13}$C NMR only with pretrained transformer, (top right) $^{1}$H NMR only with pretrained transformer, (bottom left) $^{13}$C + $^{1}$H NMR with pretrained transformer, and (bottom right) $^{13}$C + $^{1}$H NMR without pretrained transformer.

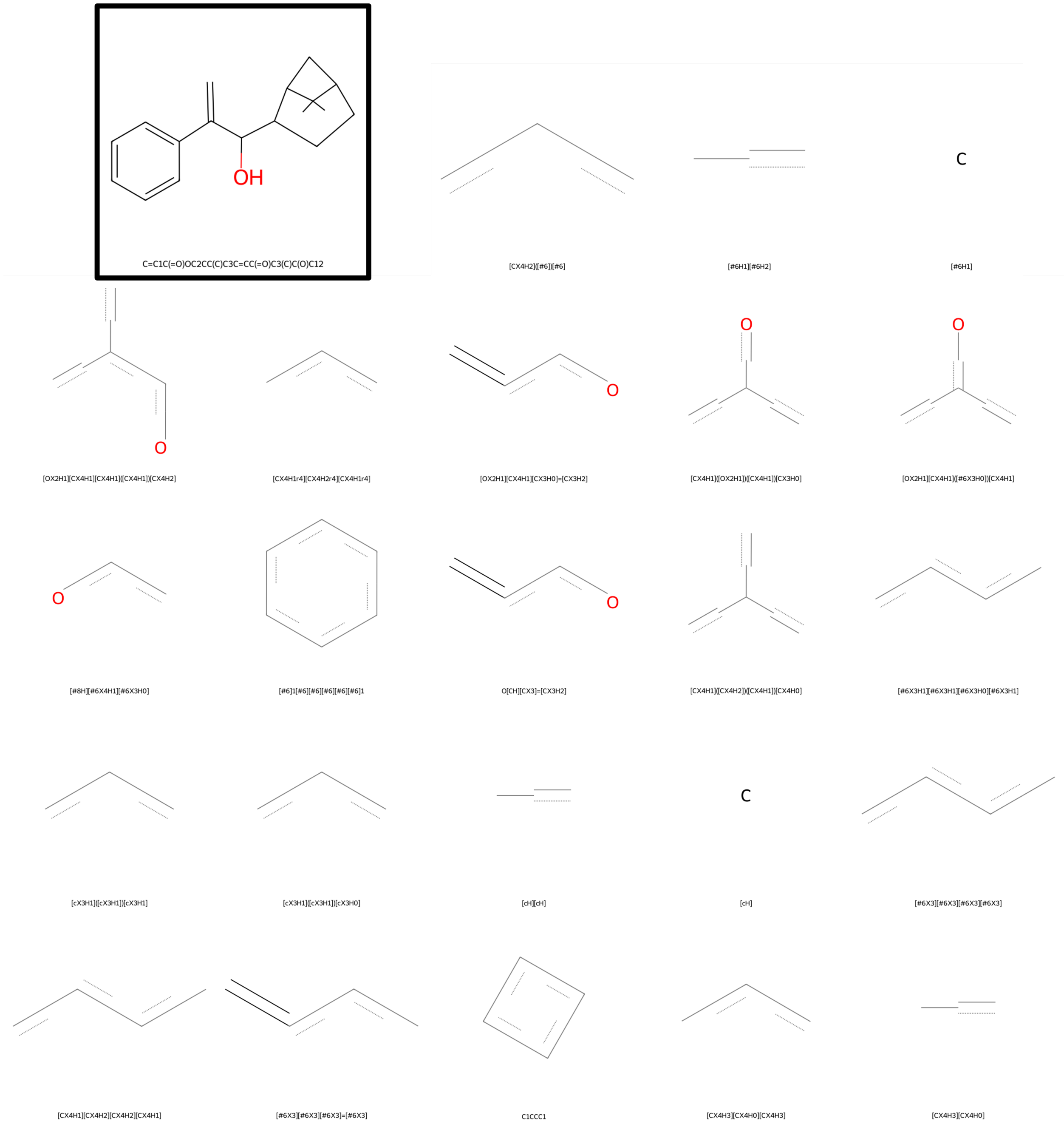


Figure 5: Example of a correctly predicted molecule (top left, black box) from the substructure-to-structure model and the first 23 of 53 substructures that were provided as input.

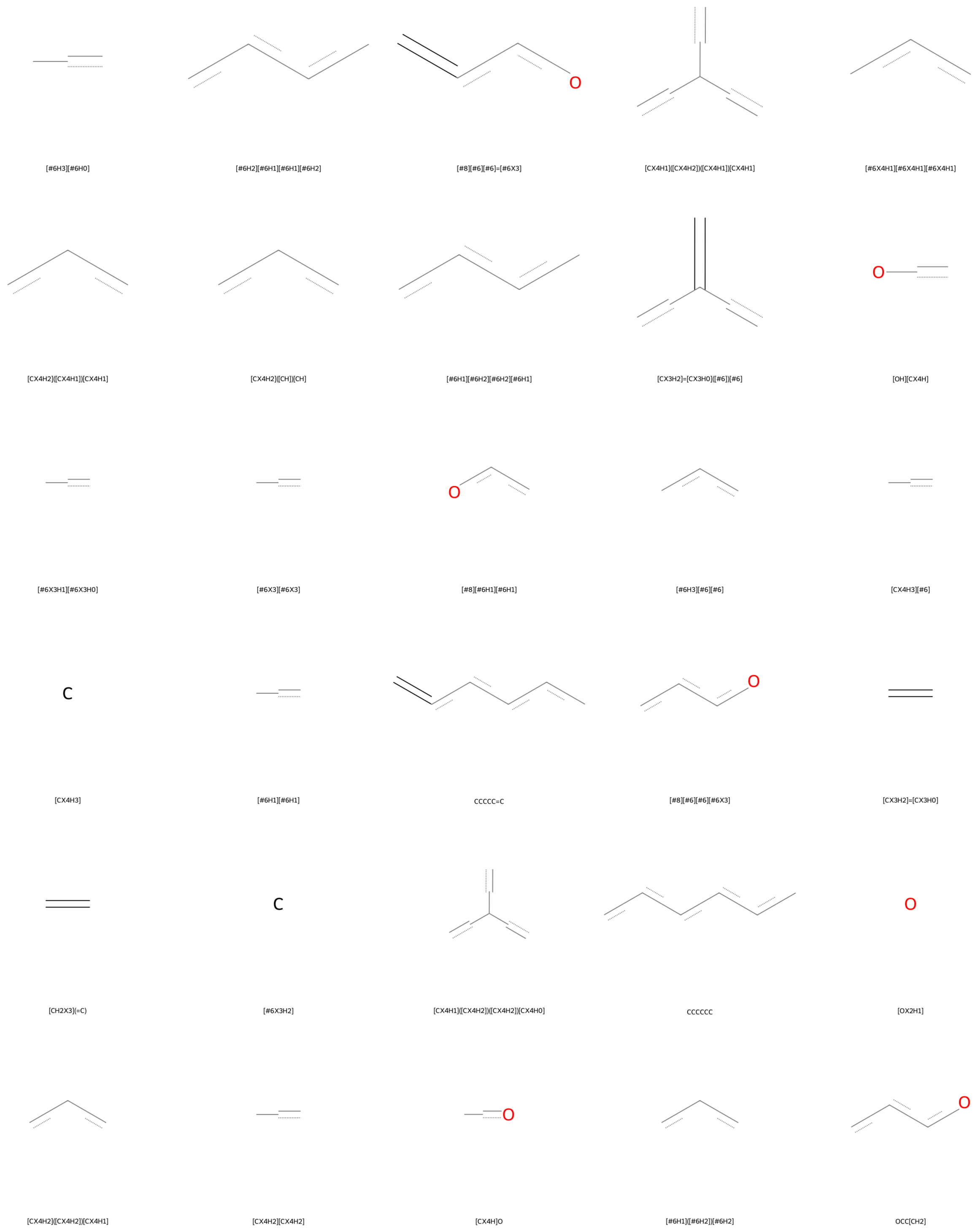


Figure 5: The remaining 30 of 53 substructures that were provided as input.

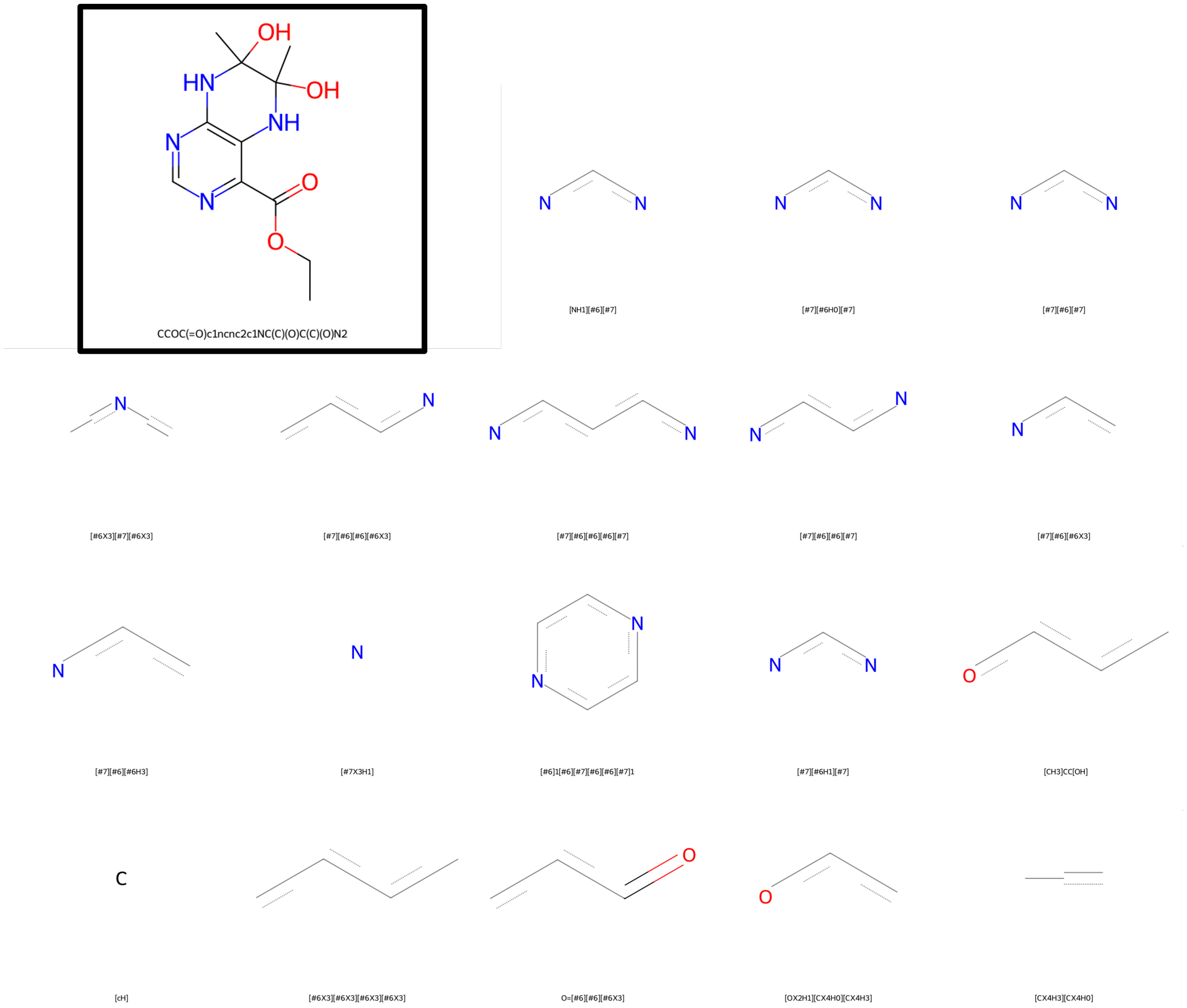


Figure 6: Example of a correctly predicted molecule (top left, black box) from the substructure-to-structure model and the first 18 of 36 substructures that were provided as input.

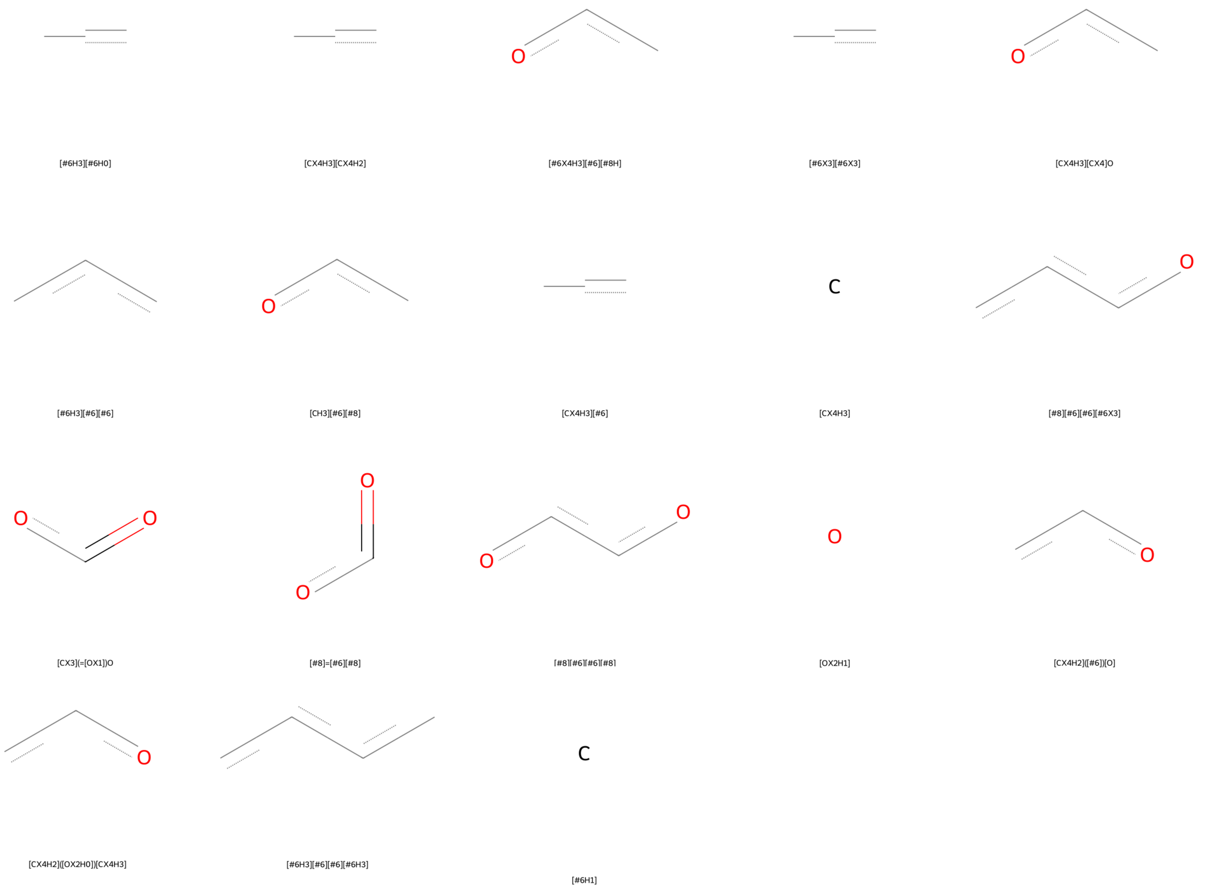


Figure 6: The remaining 18 of 36 substructures that were provided as input.

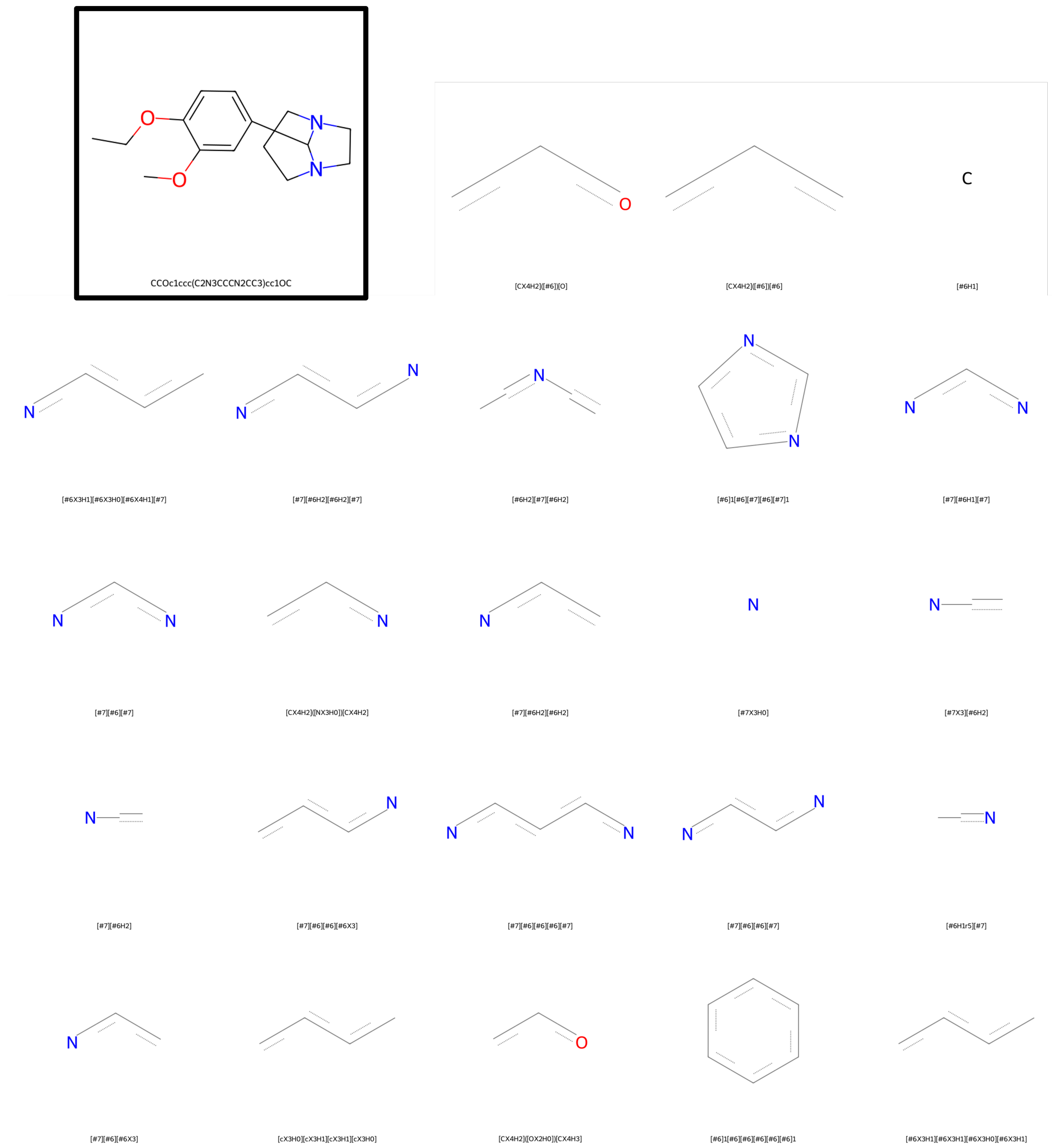


Figure 7: Example of a correctly predicted molecule (top left, black box) from the substructure-to-structure model and the first 23 of 43 substructures that were provided as input.

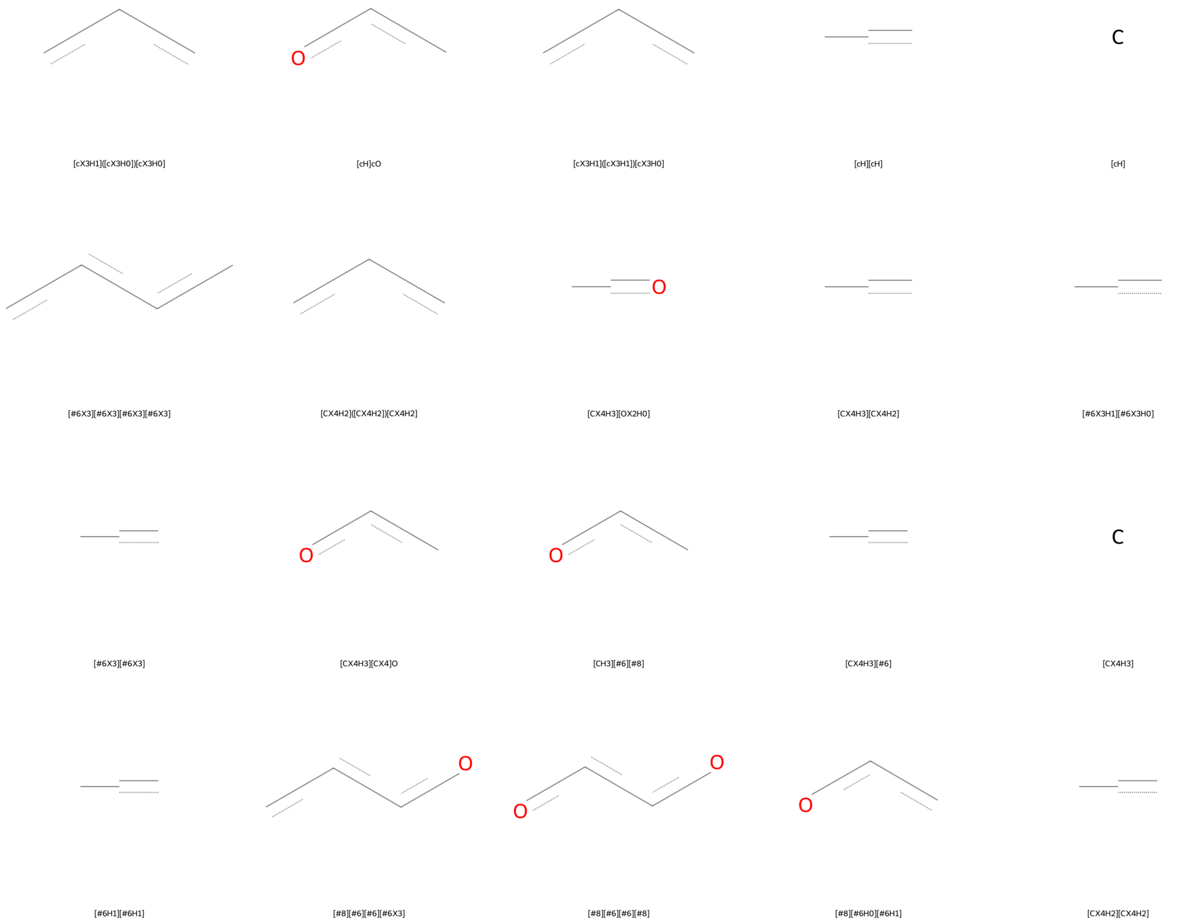


Figure 7: The remaining 20 of 43 substructures that were provided as input.

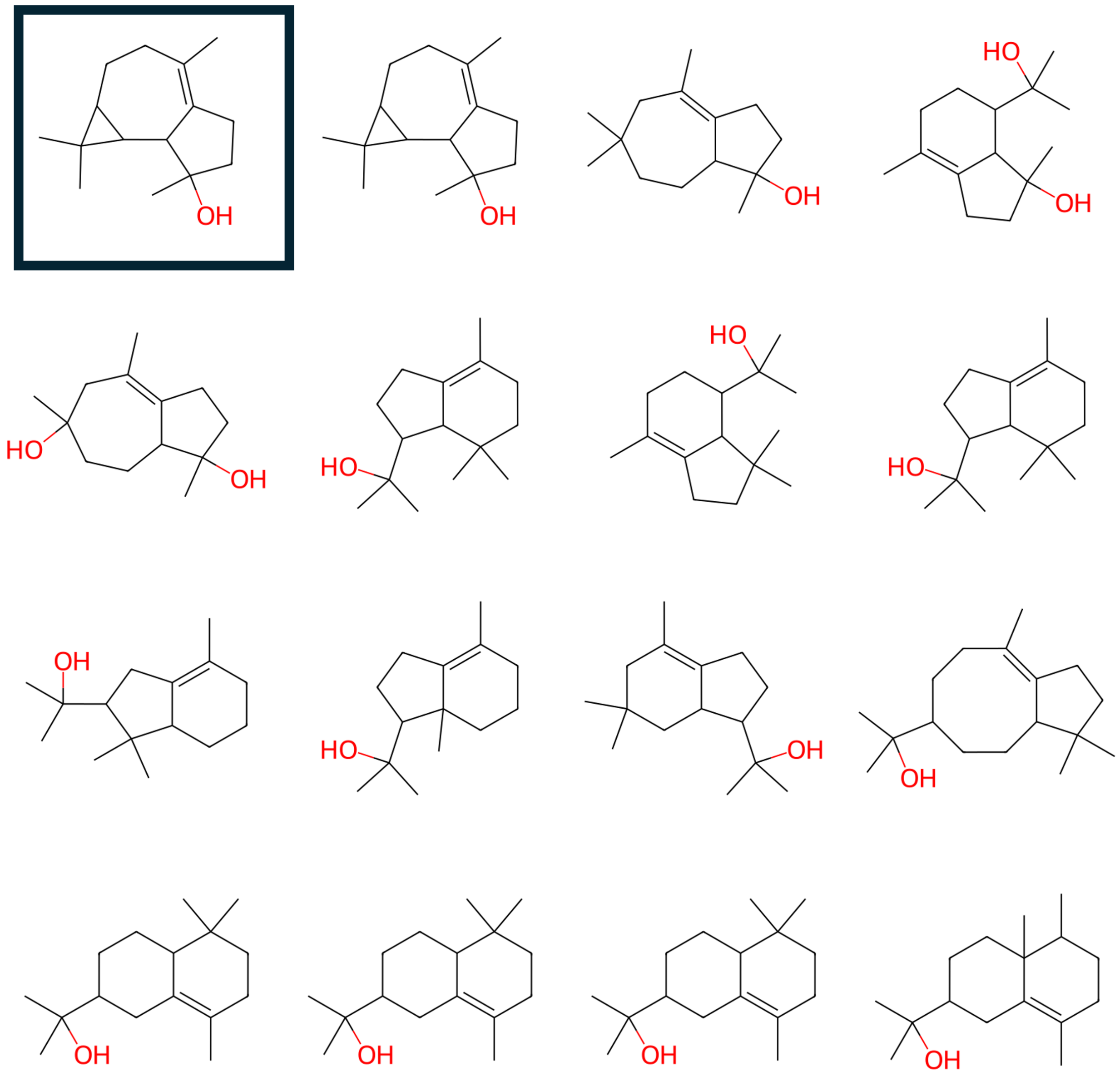


Figure 8: An example of one of the molecules correctly predicted by the substructure-to-structure transformer model (top left, black box) and all 15 structures generated by the model.

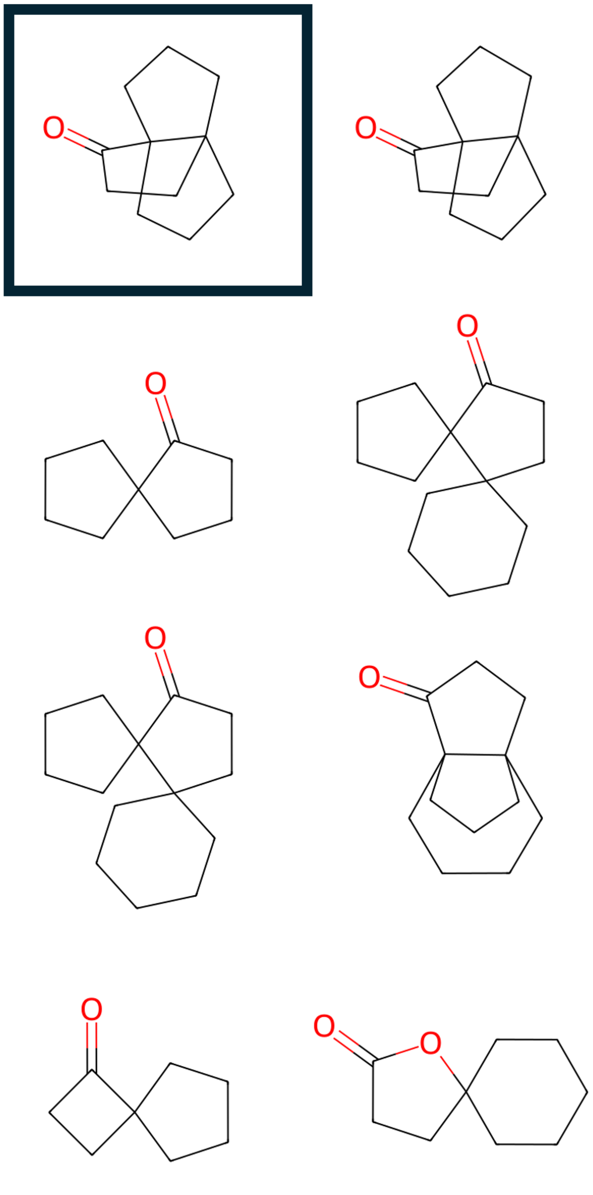


Figure 9: An example of one of the molecules correctly predicted by the substructure-to-structure transformer model (top left, black box) and all 15 structures generated by the model.

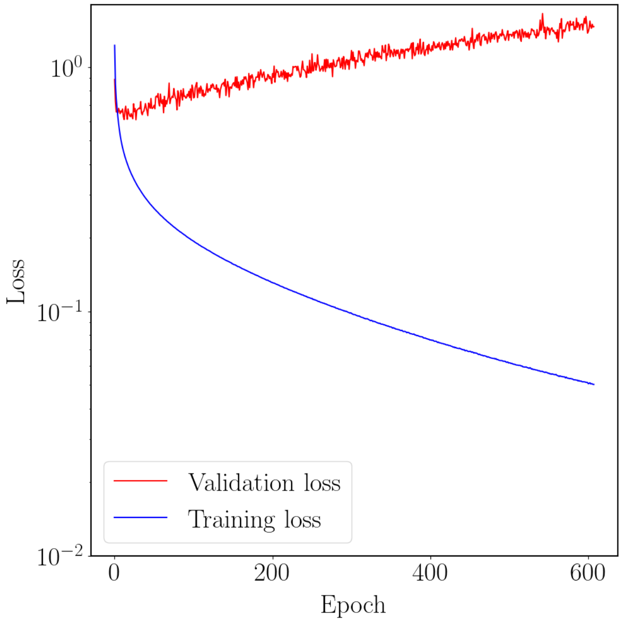


Figure 10: Training and validation loss curves for the multitask model with simulated training data and experimental validation/test data. The divergence of the training loss on simulated data and validation loss on experimental data shows clear overfitting of the model to the simulated spectra.

# References


(1) Vaswani, A.; Shazeer, N.; Parmar, N.; Uszkoreit, J.; Jones, L.; Gomez, A. N.; Kaiser, L.; Polosukhin, I. Attention Is All You Need. **2017**, Publisher: arXiv Version Number: 7.

(2) Paszke, A. et al. PyTorch: An Imperative Style, High-Performance Deep Learning Library. 2019; `http://arxiv.org/abs/1912.01703`, arXiv:1912.01703 [cs, stat].

(3) Hinton, G. E.; Srivastava, N.; Krizhevsky, A.; Sutskever, I.; Salakhutdinov, R. R. Improving neural networks by preventing co-adaptation of feature detectors. 2012; `http://arxiv.org/abs/1207.0580`, arXiv:1207.0580 [cs].

(4) Ba, J. L.; Kiros, J. R.; Hinton, G. E. Layer Normalization. 2016; `http://arxiv.org/abs/1607.06450`, arXiv:1607.06450 [cs, stat].

(5) Hassani, A.; Walton, S.; Shah, N.; Abuduweili, A.; Li, J.; Shi, H. Escaping the Big Data Paradigm with Compact Transformers. 2022; `http://arxiv.org/abs/2104.05704`, arXiv:2104.05704 [cs].

(6) John Wiley & Sons, Inc. SpectraBase. `https://spectrabase.com/`.

(7) RDKit: Open-source cheminformatics. `https://www.rdkit.org/`.

(8) Ruddigkeit, L.; Van Deursen, R.; Blum, L. C.; Reymond, J.-L. Enumeration of 166 Billion Organic Small Molecules in the Chemical Universe Database GDB-17. *Journal of Chemical Information and Modeling* **2012**, *52*, 2864–2875.

(9) Huang, Z.; Chen, M. S.; Woroch, C. P.; Markland, T. E.; Kanan, M. W. A framework for automated structure elucidation from routine NMR spectra. *Chemical Science* **2021**, *12*, 15329–15338.

(10) Schwaller, P.; Gaudin, T.; Lányi, D.; Bekas, C.; Laino, T. “Found in Translation”: predicting outcomes of complex organic chemistry reactions using neural sequence-to-sequence models. *Chemical Science* **2018**, *9*, 6091–6098.

(11) O'Boyle, N. M.; Banck, M.; James, C. A.; Morley, C.; Vandermeersch, T.; Hutchison, G. R. Open Babel: An open chemical toolbox. *Journal of Cheminformatics* **2011**, *3*, 33.

(12) Willcott, M. R. MestRe Nova. *Journal of the American Chemical Society* **2009**, *131*, 13180–13180.

(13) Kingma, D. P.; Ba, J. Adam: A Method for Stochastic Optimization. 2017; `http://arxiv.org/abs/1412.6980`, arXiv:1412.6980 [cs].
